# Factors influencing health-seeking behavior among Sudanese immigrants in Saudi Arabia

**DOI:** 10.1186/s12889-024-19122-4

**Published:** 2024-06-22

**Authors:** Noor S. Elfaki, Hafeia A. Abdelgyoum, Ala Elhelali

**Affiliations:** 1 Faculty of Medicine, University of Khartoum, Khartoum, Sudan; 2 Department of Plastic and Reconstructive Surgery, Johns Hopkins University, Baltimore, MD USA

**Keywords:** Health-seeking behavior, Healthcare-seeking behavior, Factors influencing health-seeking behavior, Sudanese immigrants, Saudi Arabia, Health insurance

## Abstract

**Background:**

Health-seeking behavior (HSB) involves any action or inaction taken by individuals who perceive themselves to have a health problem or illness aimed at finding appropriate medical treatments. Studies suggest a positive relationship between the availability and quality of health services and their utilization. This study aimed to identify the factors influencing health-seeking behavior among Sudanese immigrants in Saudi Arabia, to improve healthcare access and health outcomes.

**Method:**

A cross-sectional study was conducted targeting Sudanese residents of the Kingdom of Saudi Arabia (KSA). Participants were recruited using convenient sampling. A self-administered questionnaire was distributed electronically. A total of 494 participants were recruited for the study.

**Results:**

This study showed that the majority of the participants (66.6%) visited a primary healthcare center when faced with a medical problem. However, the prevalence of self-medication in the past three months was 45.7%. Significant factors influencing health-seeking behavior included age (OR [95% CI]: 1.032 [1.000-1.066]) and lack of health insurance (OR = 1.01, 95% CI [1.00-1.02], *p* = 0.019).

**Conclusions:**

This study emphasizes the importance of understanding healthcare-seeking behavior among immigrant groups, particularly Sudanese immigrants in Saudi Arabia. It highlights the significance of insurance as a determinant of healthcare-seeking behavior and calls for reforming current policies to reduce disparities in accessing healthcare services.

## Background

Health-seeking behavior (HSB) refers to actions taken by individuals when they perceive a health problem or illness, aiming to find an appropriate treatment option [1]. HSB is initiated by a decision-making process that is influenced by individual and/or familial behavior, community standards and norms, and provider-related features and behavior [2]. Various studies demonstrated that socioeconomic status, sex, age, social standing, type of disease, access to resources, and perceived quality of care all influence the decision to engage with a specific medical channel [3, 4]. This behavior, on an aggregate and global scale, has the potential to organize and strongly influence the demand for healthcare services [5]. 

Access to and use of treatment services are considered fundamental rights. Studies suggest that there is a positive relationship between the availability and quality of health services and their utilization [6–9]. However, inequalities in access to these services have been observed, indicating that patients from lower socioeconomic backgrounds, and certain nationalities and minority groups, benefit less from these services [10]. 

Migration defined as the movement of people across borders or within a country, presents additional challenges [11]. According to recent International Organization of Migration (IOM), there were approximately 281 million international migrants worldwide in 2020 [12]. These individuals face new social, economic, and political conditions in their host countries impacting their HSB. In particular, labor migration is associated with high stress levels, poor working conditions, and poor psychological and physical health [13]. Moreover, there is consistent evidence that immigrant groups face prejudice and have limited access to healthcare as seen in the Arab Gulf countries, where Immigrants community has less access to healthcare [14]. In Saudi Arabia, Immigrants are particularly socially disadvantaged because of their low average income and lack of knowledge local resources, struggle to access necessary health services [15]. 

The IOM classifies Sudan as a country with active migration movements both internally and internationally [16] In recent years, Sudanese migrants have primarily sought asylum in the oil-rich Gulf Arab countries [17]. According to official census data, there are 819,600 Sudanese migrants in Saudi Arabia, constituting 6.1% of the migrant population [18]. The ongoing war in Sudan has further exacerbated migration with over 1 million people fleeing the country into neighboring countries. If this conflict continues, it is estimated that almost 2 million refugees and migrants will be affected by the crisis [16, 17]. 

Sudanese immigrants face unique challenges accessing healthcare in Saudi Arabia. Cultural differences and lack of knowledge of the healthcare system are some of the barriers that can prevent them from seeking medical care. While most research on on immigrant health have focused on Western countries [19], there is a need to understand the unique challenges faced by immigrants in non-Western contexts.

The United Nation’s (UN’s) Sustainable Development Goal (SDG) 3.8, aims to “Achieve universal health coverage, including financial risk protection, access to quality essential health-care services and access to safe, effective, quality and affordable essential medicines and vaccines for all.” [20] However, immigrants tend to underutilize the healthcare services that are offered due to exclusion from local health systems [21–23]. Understanding the HSB of migrants can help achieve universal health coverage and meet SDG targets.

This study aims to identify the factors influencing health-seeking behavior among Sudanese immigrants in Saudi Arabia. By understanding these factors, policymakers and healthcare providers can develop targeted interventions to improve access to healthcare services for this population. This, in turn, can help reduce disparities in healthcare access and outcomes among Sudanese immigrants and move towards achieving universal healthcare coverage for all individuals, regardless of their nationality or immigration status.

Additionally, by promoting culturally sensitive healthcare interventions based on the findings of this study, healthcare providers can improve the overall quality of care provided to Sudanese immigrants in Saudi Arabia. This can lead to better health outcomes, increased trust in the healthcare system, and ultimately contribute to the goal of universal healthcare by ensuring that all individuals receive high-quality, equitable healthcare services.

This study fills a critical gap in the existing literature as it represents the first attempt to investigate HSB among Sudanese immigrants in Saudi Arabia. By focusing specifically on this demographic group, the research aims to provide a nuanced understanding of the unique factors influencing their healthcare utilization patterns. This approach is essential for developing culturally sensitive and tailored interventions that effectively address the healthcare needs of Sudanese immigrants in Saudi Arabia. Furthermore, the findings of this study can contribute to the broader body of knowledge on migrant health and serve as a foundation for future research in this underexplored area.

Overall, this study has the potential to inform policy decisions and healthcare practices that can help advance progress towards universal healthcare coverage, aligning with the global commitment to ensure health for all and leaving no one behind.

## Methods

### Study design and population

A community-based cross-sectional study was conducted using an online population survey. Participants were recruited using convenient sampling. Eligibility criteria included male and female Sudanese immigrants ≥ 18 years of age, residing in the Kingdom of Saudia Arabia (KSA) on a residence visa, at the time of the study. The exclusion criteria were Sudanese immigrants on a visiting visas, those coming for religious rites (Haj or Umrah), and individuals < 18 years of age. Data collection occurred between August 1st to September 1st, 2023. A total of 530 responses were collected during the study period. After excluding respondents with visiting visas, a total of 494 participants were included for analysis.

### Data collection tools

A self-administered questionnaire was developed in Arabic using Google Forms. The questionnaire was reviewed and refined by two public health experts( S.A and E.M). A pilot study involving, 30 participants was conducted to test the questionnaire, and newcessary adjustments were made. The final questionnaire consisted of two parts: the first section captured demographic information, the length of residency in the KSA, and the health insurance status. The second section assessed the participants’ HSB and consisted of four questions. The first question asked about the first action taken after encountering a health problem, and the following three questions evaluated self-medication behavior.

### Data collection method

The questionnaire was distributed electronically through Sudanese social groups on social media platforms such as Facebook and WhatsApp. This approach facilitated the efficient dissemination of the survey among the target population. Efforts were made to ensure a representative sample, by distributing the survey through multiple groups with diverse demographics in terms of age, sex, and occupation.

### Data analysis

Data analysis was conducted using SPSS (version 26.0). Frequencies and percentages, were calculated to summarize categorical variables such as the participants demographics (age, sex, martial status, duration of residency, health insurance status) and HSB (initial action taken when encountering a health problem and self medication practices). Chi-square analysis was performed to determine any correlations between sociodemographic variables andhealth-seeking behavior. To identify significant predictors of health-seeking behavior, logistic regression analysis was performed. Variables found to be significant in the chi-square tests were included in the logistic regression model. The model provided odds ratios (OR) with 95% confidence intervals (CI) to quantify the strength of associations between predictor variables and the likelihood of positive HSB. The Hosmer and Lemeshow goodness-of-fit test assessed the model fit, with a *p*-value greater than 0.05 indicating an acceptable fit. A *P* value of ≤ 0.05 was considered significant.

## Results

During the study period, a total of 494 participants were recruited. The majority of participants were males (86.6%), with a mean age of 36.5 years (+/- 9.25 years). Most participants were employed in the private sector (71.7%) and reported an average monthly income of 4467 Saudi Arabian Riyal (SAR) (+/- 4097 SAR). Nearly half of the respondents (44.1%) had no insurance or only informal insurance. Additionally, 20.3% of participants reported having a chronic disease, with hypertension (10.5%) or diabetes mellitus (7.9%) being the most common conditions (Table 1).

**Table 1:** Demographic characteristics of the studied population

Characteristic	Divisions	Frequency (*N*)	Percentage (%)
Age	< 30 years old	146	29.6
	30-50-year-old	296	60.1
	> 50-year-old	51	10.3
Gender	Male	428	86.6
	Female	66	13.4
Marital status	Single	162	32.8
	Married	322	65.2
	Divorces, separated or widowed	10	2.0
Highest educational level	Primary or secondary education	74	15.0
	Diploma or Bachelor’s degree	328	66.4
	Higher studies	92	18.6
Occupation	Government sector employee	25	5.1
	Private sector employee	354	71.7
	Nonskilled worker or crafts maker	64	13.0
	Housewife	18	3.6
	Student	7	1.4
	Unemployed or retired	25	5.1
Monthly Income in SR	0-2000 SR	134	27.1
	2000–5000 SR	235	47.6
	5000-10,000 SR	89	18.0
	10,000–30,000 SR	27	5.5
Duration of residency	< 2 years	76	15.4
	2–5 years	115	23.3
	5–10 years	131	26.5
	> 10 years	172	34.8
Chronic diseases	Yes	103	20.9
	No	391	79.1
Health insurance status	No insurance	116	23.5
	Informal insurance	100	20.2
	Silver insurance (30 K SR)	97	19.6
	Gold insurance (50 K SR)	47	9.5
	Platinum insurance (100 K SR)	35	7.1
	Diamond insurance (250 K SR)	20	4.00
	C insurance (500 K SR)	73	14.8
Total		494	100

### Health-seeking behaviour

Table 2 shows the first action taken by the respondents when they were confronted with health issues. The initial actions taken by participants when confronted with a health issue varied. The majority of respondents reported visiting a primary healthcare center (PHC), while only 3.4% taking a drug without a prescription. A total of 11.3% would self-diagnose using the internet, 10.7% claimed they would consult an acquainted doctor, and only 7.9% would consult a pharmacist.

**Table 2:** First action taken after encountering a health issue

First Action	Frequency (*N*)	Percent (%)
Go to a hospital or PHC	329	66.6
Consult a doctor from my acquaintances	53	10.7
Go to the pharmacy and consult the pharmacist	39	7.9
Try to diagnose and treat myself using the internet	56	11.3
Go and buy drugs directly without consultation	17	3.4
Total	494	100.0

### Self-medication practices

Approximately half of the respondents (54.3%) denied taking any drugs without a doctor’s prescription in the last 3 months. Among those who engage in self-medication21.5% did so once, 10.1% twice, 5.5% three times, and 8.7% more than three times in the last three months (Table 3). Moreover, participants also cited various bases for their self-medication in the past three months: doctor’s orders (63.8%), previous experience (45.5%), advice from friends or relatives (16.2%), and information found on the internet (21.2%) (Table 4).

**Table 3:** Number of times drugs were taken without a doctor’s prescription in the last three months

	Frequency (*N*)	Percentage (%)
None	268	54.3
Once	106	21.5
Twice	50	10.1
Three times	27	5.5
More than three times	43	8.7
Total	494	100.0

**Table 4:** Basis for taking drugs in the last three months

Basis for Taking Medication		Frequency (*N*)	Percentage (%)
Doctor’s orders	Yes	315	63.8
	No	179	36.2
Previous experience	Yes	225	45.5
	No	269	54.5
Friend or relative advice	Yes	80	16.2
	No	414	83.8
Information on the internet	Yes	104	21.1
	No	390	78.9
Total		494	100

### Factors influencing health-seeking behavior

Chi-squared test revealed significant associations between HSBs and several sociodemographic variables. Significant associations were found for age (χ2 (4, *n* = 494) = 16.148, *p* = 0.003), marital status [χ2 (1, *n* = 494) = 11.657, *p* = 0.001], duration of residency in Saudi Arabia [χ2 (3, *n* = 494) = 18.956, *p* < 0.001] and health insurance status [χ2 (6, *n* = 488) = 23.382, *p* = 0.001]. However, no significant associations were found between HSB and variables such as sex, occupation, monthly income, level of education, or the presence of a chronic disease, as shown in Table 5.

**Table 5:** Association between sociodemographic factors and positive health-seeking behavior

		Positive health-seeking behavior		X2 value	*P* value
		Yes	No		
Age	18–24	9	7	16.148	0.003
	25–34	135	94		
	35–44	104	44		
	45–54	53	15		
	55–65	27	5		
gender	Female	48	18	1.286	0.257
	Male	281	147		
Marital Status	Married	232	90	12.351	< 0.000
	Single	97	75		
Educational level	Primary or secondary education	49	25	2.787	0.248
	Diploma or bachelor degree	212	116		
	Higher studies	68	24		
occupation	Professional worker	258	122	1.246	0.536
	Nonskilled worker or crafts maker	40	24		
	Unemployed, Housewife, Student, or Retired	31	19		
Income	0-2000 SR	84	50	2.423	0.489
	2000–5000 SR	10	75		
	5000-10,000 SR	64	25		
	10,000–30,000 SR	17	10		
Duration of residency	< 2 years	37	39	18.956	< 0.000
	2–5 years	70	45		
	5–10 years	94	37		
	> 10 years	128	44		
Chronic diseases	Yes	75	28	2.261	0.133
	No	254	137		
Health insurance status	No insurance	61	55	23.382	0.001
	Informal insurance	62	38		
	Silver insurance (30 K SR)	68	24		
	Gold insurance (50 K SR)	39	8		
	Platinum insurance (100 K SR)	28	7		
	Diamond insurance (250 K SR)	17	3		
	C insurance (500 K SR)	50	23		

A logistic regression model further identified age and health insurance as significant predictors of HSB. The logistic regression model was statistically significant, χ2 (1 1, *n* = 494) = 42.820, *p* < 0.001, explaining 12.1% (Nagelkerke R2) of the variance in health-seeking behavior, and it correctly classified 69.4% cases. The Hosmer and Lameshow goodness of fit test indicated an acceptable fit (*p* = 0.366). Out of the 4 variables we entered into the logistic regression model, only age (OR [95% CI]: 1.032 [1.000-1.066]) and lack of health insurance (OR = 1.01, 95% CI [1.00-1.02], *p* = 0.019) were found to be significant predictors of HSB, as shown in Table 6. In contrast the duration of residency and marital status did not show significant associations with HSB.

**Table 6:** Logistic regression model

		OR	95% CI	*P* value
Age		1.032	1.000–1.066	0.049
Marital status	Single	1.00		
	Married	0.830	0.498–1.385	0.476
Health insurance	No health insurance	1.00		0.019
	Informal insurance	0.643	0.335–1.233	0.184
	Silver insurance (30 K SR)	0.877	0.446–1.723	0.703
	Gold insurance (50 K SR)	1.042	0.527–2.061	0.906
	Platinum insurance (100 K SR)	2.552	0.994–6.553	0.052
	Diamond insurance (250 K SR)	1.909	0.708–5.145	0.202
	C insurance (500 K SR)	2.486	0.634–9.747	0.192
Duration of residency in KSA	Less than 2 years	1.00		0.390
	Two to five years	1.326	0.715–2.458	0.370
	Five to ten years	1.809	0.913–3.587	0.089
	More than 10 years	1.640	0.802–3.354	0.175

## Discussion

This study provides the first comprehensive analysis of health-seeking behavior among Sudanese immigrants in Saudi Arabia. Our findings demonstrate that age and health insurance status are significant predictors of HSB, with older participants and those with health insurance being more likely to seek appropriate medical care. We found a 66.6% rate of positive HSB. These results align with those of other studies performed in Malaysia (66.7%), Nigeria (63%) and India (74.6%) [24–26]. 

### Insurance

Prior research indicates that health insurance lowers the cost of hospital stays and medication, allowing people to receive better medical care while treating various illnesses [27, 28]. However, other studies have demonstrated that insurance does not significantly increase the use of health services, particularly in situations where individuals still receive high-quality care even without insurance [29]. Nonetheless, the positive effect of insurance is more pronounced in marginalized populations [30]. 

This study revealed a strong association between health insurance status and health-seeking behavior. In this study, almost half of the participants (43.7%) had no or informal health insurance. This percentage is higher than that reported in a wider-scale study by Alkhamis et al., 2017, conducted on immigrants in Saudi Arabia, which found that only 30% were uninsured [15]. However, this discrepancy may be due to differences in demographics, as the study by Alkhamis et al., 2017, primarily included only males, non-Arab workers and did not specify the number of Sudanese participants.

Informal insurance is often considered as a way for companies and employers to evade authorities without providing workers with proper health insurance coverage. According to Saudi Arabian Labor Law, employers must bear the responsibility for paying all necessary medical expenses for their immigrant employees [31], yet many immigrant workers and their families remain uninsured.

The relationship between health insurance coverage and health-seeking behavior was corroborated by a similar study on Saudi and non-Saudi nationals in KSA. This study found that having health insurance increased the likelihood of attending a medical check-ups by approximately 13% among Saudi Arabians and 20% among non-Saudi [30]. These findings are consistent with a qualitative study on low-income Bangladeshi migrant workers in the United Arab Emirates, where participants cited insurance structures as barriers to achieving good health care [13]. 

### AGE

The positive association between age and HSB indicates that older Sudanese immigrants are more likely to seek appropriate healthcare when faced with medical issues. This could be attributed to greater health awareness and the higher prevalence of chronic disease among older individuals, necessitating more frequent interactions with healthcare providers. In addition, older Sudanese immigrants are more likely experts in their professions and have better access to higher categories of health insurance. This positive association was similarly described in a study in Senegal that found an increase in the odds of seeking healthcare among people older than 40 years [9], contradicting the findings of another study conducted in Iran that revealed an inverse relationship between age and appropriate HSB, where older people were less likely to seek healthcare [10]. The practical implications of these findings suggest a need for targeted health education campaigns aimed at younger immigrants to promote proactive HSBs and early intervention. These campaigns could focus on raising awareness about the importance of regular medical check-ups and managing chronic conditions to improve overall health outcomes.

### Monthly income

Although the association with insurance was significant, monthly income was not. This is contrary to the belief that better income will instantly mean better access to healthcare and affordability of health services. However, this finding aligns with that of another study that was conducted on both Saudi Arabian and non-Saudi Arabians in Al-Ahsa, which found that perceived economic status, rather than actual monthly income, significantly influenced HSB [32].

We believe that the immigration status of our participants may have influenced these results. Unlike Saudi Arabian citizens, non-Saudi Arabian citizens do not have access to free medical care in governmental facilities. Consequently, immigrants must obtain either health insurance or, if uninsured cover medical cost through out-of-pocket payments to receive medical care [30]. Nonetheless, the average monthly income for Saudi Arabians is approximately 10,000 SAR, and 94.5% of our participants had a monthly income below this average (4500 SAR). This disparity suggest that even a modest increase in monthly income for immigrants do not substantially impact their healthcare decisions of compared to the availability of insurance, which provides significantly more affordable healthcare services, even at its most basic levels. Therefore, enhancing health insurance coverage for expatriates may be a more effective strategy to improve healthcare access than merely increasing income levels.

### Negative HSB

Despite a relatively high percentage of positive HSB in our sample, 45.7% of the participants practiced self-medication without a doctor’s prescription. This is a high percentage compared to that in other countries such as Malaysia (11.8%) [24]. However, it is considerably lower than the rate in Sudan. A recent study by Isameldin et al., 2020, reported the prevalence of self-medication In Sudan was 100% [33]. Another study assessed self-medication with antibiotics in Sudan and reported a rate of 87% [34]. These findings highlights the importance of healthcare system affordability and health insurance in promoting positive HSB, as well as the necessity for strict regulations to prevent pharmacists from dispensing medication without prescriptions.

Unexpectedly, very few of our participants reported using the Internet as a source of health and treatment information. This finding contrasts sharply with another study by Almuammar et al., 2021. Almuammar et al., 2021 found that 92.6% of participants in KSA used social media as a source of medical information [35]. Despite the lack of significant research discussing the online HSB of the Sudanese population, the low rate observed in our study suggests a significant gap. While this low rate of internet use might protect against misinformation and inappropriate self-medication, it also means that Sudanese immigrants are missing out on valuable online health awareness campaigns and health education resources. Therefore, efforts to integrate culturally appropriate online health education and awareness initiatives could enhance overall health outcomes for this population.

### Implications

The findings of this study highlight health insurance as a crucial modifiable risk factor for negative health-seeking behavior. With 71% of our sample employed in the private sector, the lack of insurance coverage for employees and their families emerges as a significant issue contributing to suboptimal HSB among Sudanese immigrants. This inadequacy has detrimental effects on the well-being of these individuals. Therefore, urgent action is needed to enforce the Saudi Arabian labor law effectively to safeguard the health rights of vulnerable immigrant populations.

Furthermore, implementing interventions such as offering subsidized healthcare services in primary care facilities, maternal health, and mental health services could address the barriers faced by immigrants who are legally restricted from obtaining or maintaining health insurance. These targeted initiatives have the potential to improve access to essential healthcare services and enhance the overall health outcomes of immigrant communities.

### Strength and limitations

As the first paper to be written on the health-seeking behavior of Sudanese immigrants, our study provides a foundation for future research and programs aimed at improving the health status and healthcare access of Sudanese immigrants and refugees worldwide. With the ongoing war in Sudan leading to a surge in refugees, our findings are particularly relevant and timely.

The limitations of our study primarily stem from the use of a convenient sampling technique. Limited resources compelled us to gather data solely from Sudanese social groups on Facebook and WhatsApp, resulting in a higher proportion of middle-aged male workers in our sample. Consequently, our study may have inadvertently excluded other segments of the population, such as females and young males. To address this limitation, we recommend that future research be conducted on a community or household basis to ensure a more representative sample and enhance the accuracy of the findings. It is important to note that the under-representation of females in our sample may also be attributed to the lower percentage of females among Sudanese immigrants and the low response rate in females-only Facebook and WhatsApp groups. Thus, there is a need for further investigation to explore the impact of gender on health-seeking behavior.

Furthermore, the cross-sectional design employed in this research captures a snapshot of the participants’ experiences at a specific point in time, limiting their ability to establish causal relationships or discern temporal patterns. Longitudinal studies would offer a more comprehensive understanding of how these factors evolve among the immigrant population.

Our study focused on health-seeking behavior toward any illness or medical issue, which may not fully capture the nuances of behavior toward specific illnesses. Factors such as severity and duration of the condition could potentially influence individuals’ responses and actions differently. Moreover, the retrospective nature of our study introduces the possibility of recall biases, as participants may not accurately remember past health-seeking experiences. Additionally, response bias cannot be ruled out, as participants may have provided answers that they perceived as more socially desirable. These limitations underscore the need for caution when interpreting our findings and highlight the importance of further research to explore these aspects in more detail.

## Conclusion

Understanding the determinants of HSB among immigrant groups is crucial for improving healthcare access and resource allocation for this population. As inappropriate HSB has been linked to poorer health outcomes, identifying the factors that influence HSB can provide valuable insights into designing tailored health interventions to support the Sudanese immigrant community in KSA.

This study highlights the significance of insurance as a determinant of HSB, emphasizing the need for reforming current insurance policies that prioritize private companies over the health of immigrants. Further research is needed to explore the healthcare preferences and behaviors of Sudanese people and other migrants, particularly women and young people, to develop targeted policies aimed at reducing socioeconomic disparities in accessing healthcare services. By promoting universal health coverage, these policies can contribute to achieving sustainable development goals and improving the overall health and well-being of immigrant populations.

## Data Availability

The datasets used and/or analyzed during the current study are available from the corresponding author upon reasonable request.

## Abbreviations

HSB: Health-seeking behavior
KSA: Kingdom Saudi Arabia
SDG: Sustainable development goal
IOM: International organization of migration
KSA: Kingdom of Saudi Arabia
SAR: Saudi Arabian Riyal
UN: United Nations
PHC: Primary health care
